# Taurolidine Antiadhesive Properties on Interaction with *E. coli;* Its Transformation in Biological Environment and Interaction with Bacteria Cell Wall

**DOI:** 10.1371/journal.pone.0008927

**Published:** 2010-01-28

**Authors:** Francesco Caruso, James W. Darnowski, Cristian Opazo, Alexander Goldberg, Nina Kishore, Elin S. Agoston, Miriam Rossi

**Affiliations:** 1 Istituto di Chimica Biomolecolare, Consiglio Nazionale delle Ricerche, Rome, Italy; 2 Department of Medicine, Division of Hematology/Oncology, Rhode Island Hospital and Brown University, Providence, Rhode Island, United States of America; 3 Academic Computing Services, Vassar College, Poughkeepsie, New York, United States of America; 4 Accelrys, Inc., San Diego, United States of America; 5 Department of Chemistry, Vassar College, Poughkeepsie, New York, United States of America; Deutsches Krebsforschungszentrum, Germany

## Abstract

The taurine amino-acid derivative, taurolidine, bis-(1,1-dioxoperhydro-1,2,4-thiabiazinyl–4)methane, shows broad antibacterial action against gram-positive and gram-negative bacteria, mycobacteria and some clinically relevant fungi. It inhibits, *in vitro,* the adherence of *Escherichia coli* and *Staphylococcus aureus* to human epithelial and fibroblast cells. Taurolidine is unstable in aqueous solution and breaks down into derivatives which are thought to be responsible for the biological activity. To understand the taurolidine antibacterial mechanism of action, we provide the experimental single crystal X-ray diffraction results together with theoretical methods to characterize the hydrolysis/decomposition reactions of taurolidine. The crystal structure features two independent molecules linked through intermolecular H-bonds with one of them somewhat positively charged. Taurolidine in a biological environment exists in equilibrium with taurultam derivatives and this is described theoretically as a 2-step process without an energy barrier: formation of cationic taurolidine followed by a nucleophilic attack of O(hydroxyl) on the exocyclic C(methylene). A concerted mechanism describes the further hydrolysis of the taurolidine derivative methylol-taurultam. The interaction of methylol-taurultam with the diaminopimelic NH_2_ group in the *E. coli* bacteria cell wall (peptidoglycan) has a negative ΔG value (−38.2 kcal/mol) but a high energy barrier (45.8 kcal/mol) suggesting no reactivity. On the contrary, taurolidine docking into *E. coli* fimbriae protein, responsible for bacteria adhesion to the bladder epithelium, shows it has higher affinity than mannose (the natural substrate), whereas methylol-taurultam and taurultam are less tightly bound. Since taurolidine is readily available because it is administered in high doses after peritonitis surgery, it may successfully compete with mannose explaining its effectiveness against bacterial infections at laparoscopic lesions.

## Introduction

Taurolidine is an anti-infective drug with a broad spectrum of activity against gram-positive and gram-negative bacteria, anaerobic organisms and fungi, which is used clinically to prevent infections after peritonitis surgery [1]–[2]. Jacobi [3] showed that taurolidine is also effective in attenuating tumor spread that normally results after laparoscopic tumor removal [4]. A model study done with incubation of adenocarcinoma cells along with taurolidine and/or heparin in rats showed decreased amount of tumors *in vivo* and significant decrease of tumor cells *in vitro*; not only was the effect of taurolidine stronger than that of heparin but, in addition, taurolidine led to diminished E-cadherin production [5]. More recently, the antineoplastic activity of taurolidine has been shown against primary [6]–[9] and metastatic tumors [10]–[14]. Clinical cases include glioblastoma [14]–[15], gastric cancer-recurrence [16], and ovarian cancer resistant to conventional treatment [17]. Nevertheless, anti-infective properties of taurolidine are very important. For instance, *i.v.* taurolidine was successfully applied to 11 cancer patients having non-responding microbial infections [18]. Taurolidine is effective against *Staphylococcus epidermidis* endophthalmitis on infected rabbit's eye [19], infections from vancomycin-resistant *Enterococcus faecalis* and methicillin-resistant *Staphylococcus aureus* [20]. Taurolidine also has antiendotoxin properties as it is able to abolish the lethal effect of toxins from *E. coli* in infected mice and rabbits [21]–[22]. Since the mechanism of taurolidine antiadherence activity is not known, we focus, in this study, on clarifying the molecular features responsible for this feature.

**Figure 1:**
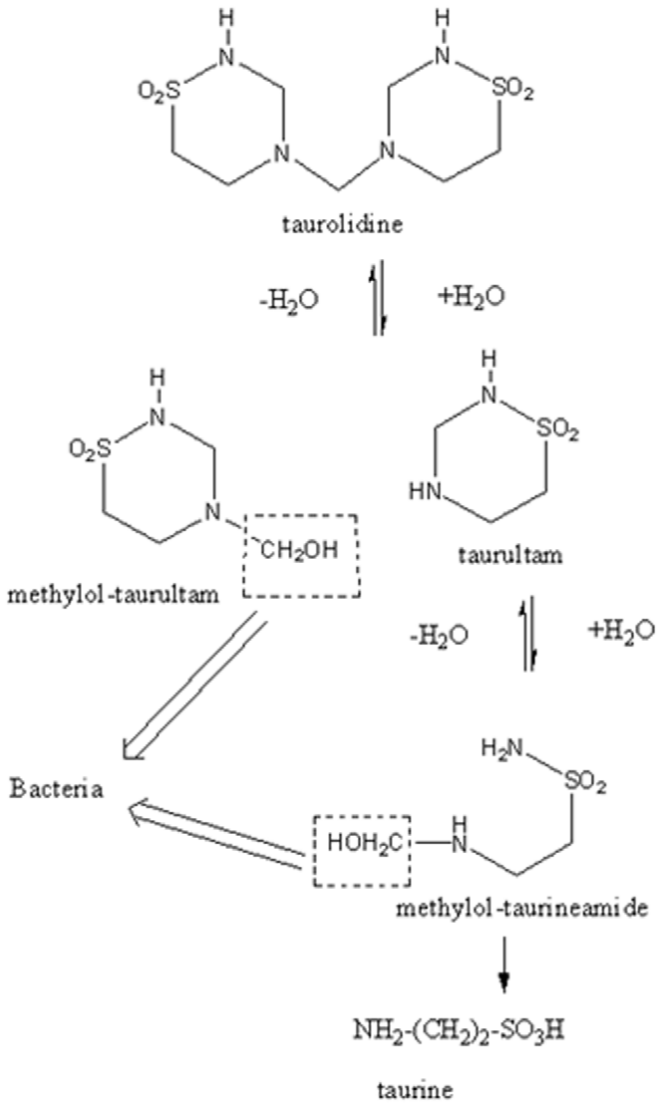
Hydrolysis/decomposition of taurolidine.

Taurolidine mechanism of action is necessarily impacted by its easy hydrolysis [23], Figure 1, in aqueous solution; this results in the production of several metabolites and earlier findings suggested that the methylol-containing species that are produced during taurolidine hydrolysis could interact at the bacterial cell wall [24]. *E. coli*, a gram-negative bacterium that colonizes the intestinal tract and lives in symbiosis with its host is a recurrent source of infection in humans. Over 80% of all urinary tract infection are caused by *E. coli* and an overwhelming majority of these uropathogenic isolates express filamentous organelle called fimbriae [25]. This system provides an excellent model to study the mechanism of action of taurolidine, since bacterium adherence is associated with F1 fimbriae and structural features of the proteins in pili are known. *E. coli* pili contain FimA (about 95%) and the minor subunits FimF, FimG and FimH. It was found that bacterial colonization is uniquely induced by FimH mediated specific adhesion to α-mannoside sites which are found in many mammalian and avian tissues. Thus, we performed docking studies of taurolidine and its derivatives on *E. coli* FimH protein and describe the results below.

The techniques we use are single crystal X-ray diffraction for the determination of 3-D taurolidine crystal and molecular structure, DFT theoretical calculations to study the hydrolysis/decomposition mechanism of taurolidine and its interaction with bacteria cell wall, and ligand-protein docking to analyze taurolidine antiadhesion properties on *E. coli* FimH.

## Results and Discussion

### a) X-Ray Diffraction Study

**Figure 2:**
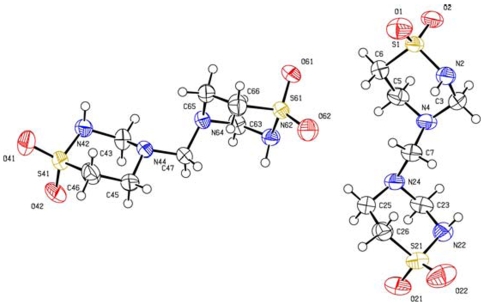
Molecular structure of the 2 independent molecules of taurolidine in the asymmetric unit of the crystal.

Figure 2 depicts the molecular structure of the 2 independent molecules in the asymmetric unit of taurolidine in the crystal. These molecules differ only in rotations around certain bonds. Table S1, deposited, shows selected geometrical features of both taurolidine molecules in the crystal. Intermolecular H-bonds are due to N(amine)-H --- O-S(sulfonyl). The packing shows one molecule having more H-bonds than the other, in one molecule they are O2 --- N62^1^* (2.92 Å), O21 --- N42^2^* (2.88 Å), O22 --- N2^3^* 2.99 (Å), N2 --- O22^3^* (2.99 Å) and N22 --- O41^4^* (3.13 Å), in the other molecule they are N42 ---O21^5^* (2.88 Å) and N62 --- O2^6^* (2.92 Å); n* indicates other molecular units. Since one independent molecule has O(sulfonyl) acting as a hydrogen bond acceptor, and the second independent molecule lacks this feature, the former (to the right in Figure 2) has somewhat more of a positive charge. It is interesting to note that the atoms involved in intermolecular hydrogen bonding are the same as those forming similar types of hydrogen bonds with amino acids in protein FimH docking studies as will be shown later.

Taurolidine molecular structure can be compared with 2,4,7-trimethyl-2,3-dihydro-4H-pyrido(4,3-e)-1,2,4-thiadiazinium 1,1-dioxide iodide [26] since they share the same atoms and connectivity in one ring. This compound has a shorter C = C bond (1.41 Å), giving it a half-chair conformation, in comparison with taurolidine's single bond C-C (1.51-1.54 Å) and chair conformations. Another closely related compound is 1,1-dioxothiane that has S-O, S-C and C-C bond lengths quite similar to those in taurolidine [27]. No unusual structural features are found comparing taurolidine with other structurally related compounds in the Cambridge crystallographic database (CSD).

### b) Decomposition of Taurolidine in Biological Environment

**Figure 3:**
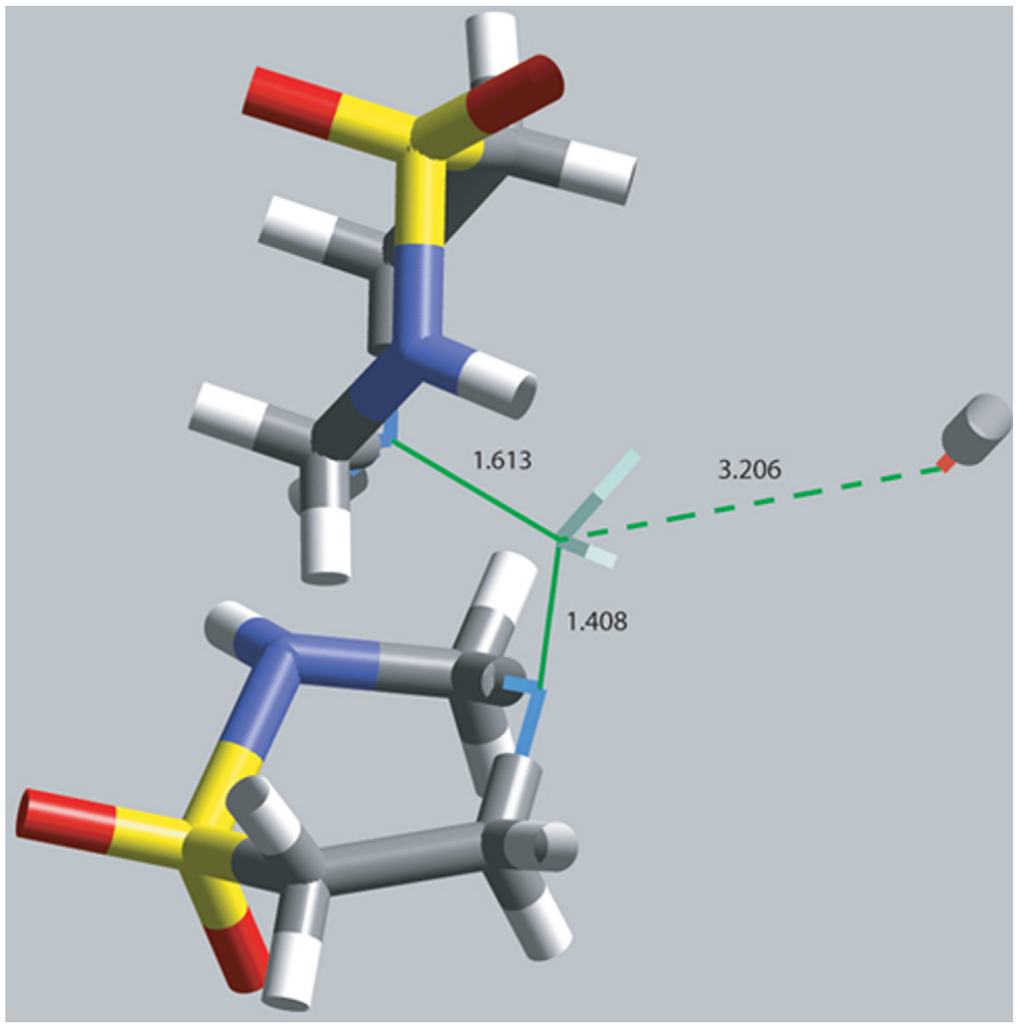
The approach of OH^−^ towards cationic taurolidine. Atoms involved in a potential transition state (TS) are wireframe style; S =  yellow, N =  blue, C =  grey, H =  white.

Taurolidine undergoes a series of hydrolysis reactions that we study theoretically. First we analyze both independent molecules in the crystal lattice with DFT methods using DMol3 (Cerius2) and, as expected, find little difference of internal energy between them (ΔE = 0.6 kcal/mol). Using the crystal coordinates of taurolidine the first hydrolysis (Figure 1) showed limited space for a non hindered approach of water to cleave the exocyclic C-N bond and, even after modifying rotational bond angles of taurolidine to achieve more clearance, we could not find a reaction path. Allowing the hydrolysis reaction to occur in 2 steps, however, showed thermodynamic and kinetic feasibility. First, we performed a H^+^ attack (using [H_3_O]^+^ or [H_5_O_2_^+^]) on the N atom that is to be cleaved from C; this process has no energy barrier and the corresponding geometry optimized cationic taurolidine shows one C(exocyclic)-NH_2_ bond (1.613 Å) longer than the other C(exocyclic)-NH bond (1.408 Å). The 2^nd^ step involved a nucleophilic attack of OH^−^ on C to cleave the lengthened C-N bond; the initial state for this reaction is depicted in Figure 3.

**Figure 4:**
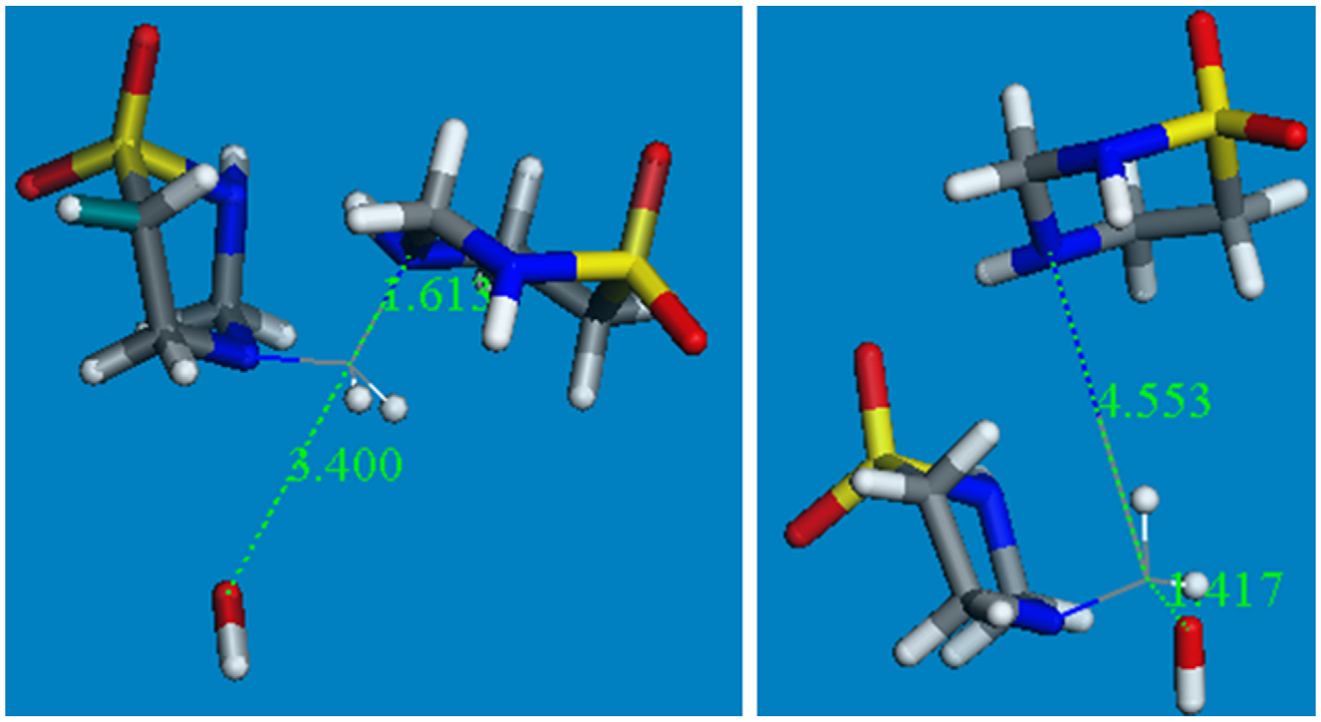
Geometry optimization for OH^−^ attack on cationic taurolidine. This is performed with DMol3 in Materials Studio: initial (left) and final (right) states.

Next, a high temperature quantum-mechanical simulation was performed to induce bond vibration to overcome potential energy barriers, while the O(hydroxyl) was directed towards the exo-cyclic C(methylene). The energy profile of the system, along with the variation of relevant geometrical parameters is shown in Figure S1. A potential transition state was not found. Therefore the geometry optimized taurolidine cation and optimized hydroxyl were placed at van der Waals distance for C and O (3.40 Å). The corresponding geometry optimization was performed and led to cleavage without TS, confirming the previous simulation. Figure 4 shows initial and final structures of this process. ΔG_Reaction_ (−109.5 kcal/mol) indicates this reaction is therefore thermodynamically feasible. The same result was obtained with Hartree-Fock at 6-31-G** level of theory.

**Figure 5:**
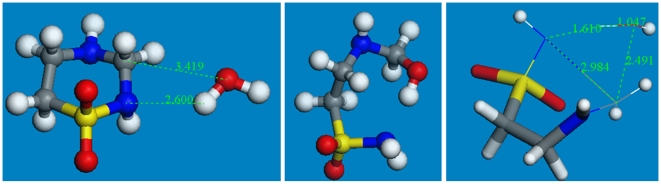
A concerted mechanism of taurultam hydrolysis. Initial state (left), final state yielding methylol-taurineamide, (center), and the transition state (right).

A related treatment for taurultam showed that, similar to taurolidine, hydrolysis is not induced by a nucleophilic attack of O(water) on C(methylene) nor from the approach of H(water) to N. In this case, a concerted mechanism was verified performing a linear synchronous transit search process. DMol3 Materials Studio about the C-N cleavage was verified for water and taurultam poised at van der Waals distance as the initial state, and methylol-taurineamide was the cleaved product (Figure 5). The found TS was associated with a unique imaginary frequency later confirmed through an optimization transition state calculation (−182 cm^−1^). Thermodynamic data are ΔG_Reaction_ of −15.1 kcal/mol and E_Activation_ of 15.4 kcal/mol and so this reaction is thermodynamically and kinetically feasible.

We also tested the 2-step mechanism for taurultam hydrolysis to compare with that of taurolidine. We confirmed that the first step, attachment of H^+^ on N, has no energy barrier. The 2^nd^ step, nucleophilic OH^−^ attack on C(methylene), was calculated at Hartree-Fock 6–31G** level of theory and the ΔG_Reaction_ of −229.4 kcal/mol indicated thermodynamic feasibility. When performing a TS search we obtained the product, suggesting no energy barrier. Therefore, a geometry minimization was submitted but the product was not obtained; these attempts failed because O(hydroxyl) ended up close to H atoms in CH_2_, NH_2_ or NH, and so this mechanism seems uncertain. Which of these 2 processes (the concerted or the 2-step hydrolysis) prevails? A feature favoring the concerted mechanism is the much higher concentration of water molecules at pH = 7 in aqueous solution (55 M) compared to [H^+^] and [OH^−^] = 10^−7^ M in the 2-step mechanism. On the contrary, the TS barrier of 15 kcal/mol in the concerted mechanism is not favored compared to no TS for the 2-step hydrolysis, but it is achievable at room temperature. Last, calculated ΔG_Reaction_ values indicate the 2-step hydrolysis is favorable. Even though the 2-step mechanism cannot be completely ascertained, the 2^nd^ hydrolysis in Figure 1 was shown to be theoretically feasible.

### c) Interaction with Peptidoglycan

The bacterial cell wall is formed by a polymeric network of saccharide-peptide units called peptidoglycan. The penicillin antibacterial mechanism involves peptidoglycan synthesis as it inhibits an enzyme that builds up the cell wall. Earlier studies suggested peptidoglycan could be a potential target for taurolidine as well [24]. A peptidoglycan monomer unit consists of two joined amino sugars, N-acetylglucosamine (NAG) and N-acetylmuramic acid (NAM), and a penta-peptide coming off of the NAM. In *E. coli,* the pentapeptide is made up of L-alanine, D-glutamic acid, *meso* diaminopimelic acid, and two D-alanines. In other bacteria, lysine replaces diaminopimelic acid, with an extra COOH.

**Figure 6:**
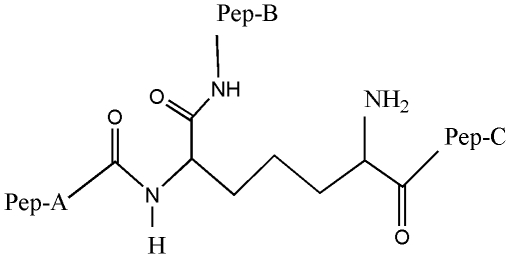
Diaminopimelic moiety in the open peptidoglycan is shown. Pep-A stays for a D-alanine moiety, Pep-B for the D-glutamic moiety, Pep-C represents a cross-linked peptide. We are testing whether methylol derivatives of taurolidine (RCH_2_OH) attack the NH_2_ group to establish a NHCH_2_R terminal moiety as shown in Figure 7.

**Figure 7:**
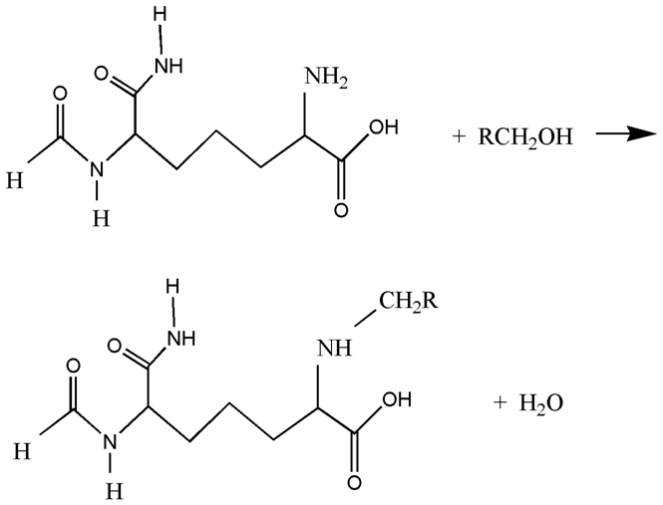
Theoretically studied reaction of a taurolidine-methylol derivative RCH_2_OH and the pimelic NH_2_ group in open peptidoglycan.

The affinity of formaldehyde for NH_2_ groups of amino acids [28] and proteins [29], through its related methylol derivatives, is useful in vaccine production. Since taurolidine metabolism produces formaldehyde [30], we study the potential interaction of methylol-taurolidine derivatives with amino acids of peptidoglycan. In particular, our interest is focused upon the action of bacterial autolysin enzymes during the growth of the cell wall, when the dipeptide cross-linking involving the diaminopimelic moiety R′-(CH_2_)_3_-CHNHR″-C(O)-R′″ becomes temporarily cleaved. This results in formation of R′-(CH_2_)_3_-CHNH_2_C(O)-R″′ and HOR”. The opening of the cell wall is associated with a process needed for cell wall renovation by the arrival and insertion of new saccharide-pentapeptide units. We hypothesize that, as with formaldehyde, R-CH_2_OH methylol groups from taurolidine will interact with the NH_2_ group of R′-(CH_2_)_3_-CHNH_2_-C(O)-R″′ to form R′-(CH_2_)_3_-CHNHCH_2_R-C(O)-R″′; a related model for the open peptidoglycan is shown in Figure 6. In our quantum-mechanical study, we use a model derived from Figure 6, namely, Pep-A and Pep-B are replaced with H atoms and Pep-C with OH. The reaction involving this model is shown in Figure 7.

**Figure 8:**
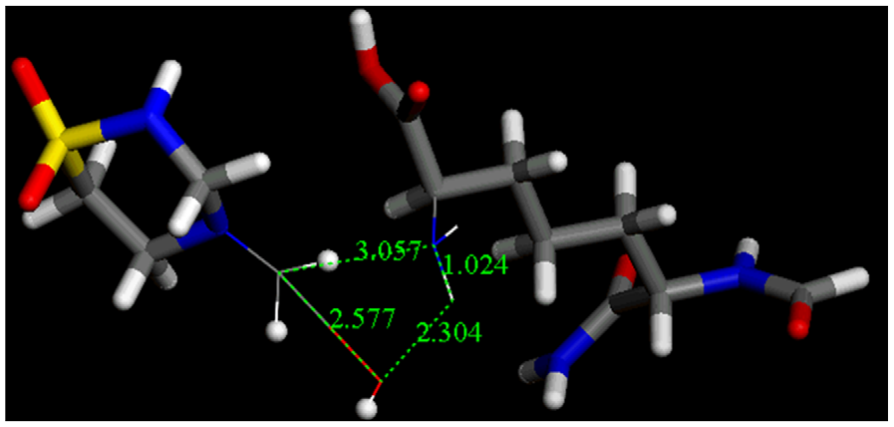
Transition state for condensation between methylol-taurultam and a diaminopimelic acid model implying water release.

This reaction is thermodynamically feasible for methylol-taurultam (ΔG = -38.2 kcal/mol), however its transition state, shown in Figure 8, has high activation energy (E_activation_ = 45.8 kcal/mol) and we conclude that, without catalysis, this reaction most likely will not occur.

Interaction between methylol-taurineamide, shown at the bottom of Figure 1, and an amine model did not indicate reactivity as ΔG resulted positive (7.5 kcal/mol).

**Figure 9:**
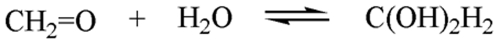
Formaldehyde hydration equilibrium.

As mentioned above, one explanation for taurolidine's mode of action has been ascribed to its production of formaldehyde. Given that formaldehyde reversibly adds water rapidly at room temperature, there are indications of equilibrium equivalence between formaldehyde and its hydrate, which is the simplest methylol species. Therefore, we explored the formaldehyde reaction with the diaminopimelic acid model and found it thermodynamically feasible. We then analyzed the equivalence between both reagents according to Figure 9.

At Hartree-Fock 6–31G** level of theory the formaldehyde hydrate is more stable (ΔG = −14.6 kcal/mol). Therefore, the interaction of taurolidine-methylol derivatives with peptides cannot be ascribed to an “aldehyde intermediate” as the latter is less stable than the former, that is, methylol will not transform into an aldehyde. Moreover, to convert methylol derivatives of taurolidine to an aldehyde, a reduction reaction is needed. From the results of all these studies, we conclude that there is no evidence of interaction between taurolidine and peptidoglycan.

### d) Antiadhesion Properties of Taurolidine

We focus now our attention on adhesion properties of bacteria. Taurolidine is active against *E. coli* [31]–[32], reducing adherence of *E. coli* bacteria to human epithelial cells in vitro [33]. Fimbriae proteins are considered important components in cell adhesion. FimH is a minor component in the Fimbriae *E. coli* protein unit and is crucial for *E. coli* infection. For instance, after directing antibodies at the FimH amino terminal [34], or inactivating the FimH gene cluster, *E. coli* lost its mannose sensitive binding activity [35]. Moreover, by direct binding of FimH to D-mannose attached to a carrier protein, it was demonstrated that this protein was uniquely responsible for the receptor specificity [36]. These results extend to other enterobacteria as well, and demonstrate that FimH is antigenically conserved in other bacterial species [37]. In addition, a substantial homology (>99%) of FimH among strains of animal (bovine, porcine and avian) was observed, although avian strains had more frequent specific mutations [38]. The role of FimH protein is to establish a bacterial anchorage to mannoside receptors in cells for later colonization [39]. The binding of the monosaccharide alpha-D-mannose is the primary bladder cell receptor for uropathogenic *E. coli* and this event requires a highly conserved FimH binding pocket [40].

**Figure 10:**
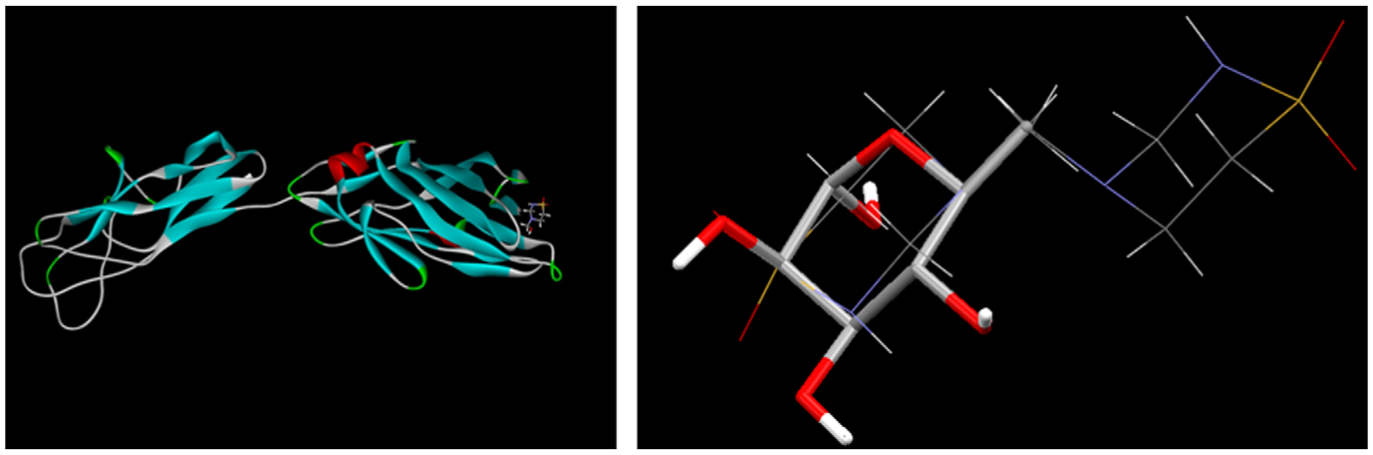
Similar structural features of taurolidine and mannose. Left: *E. coli* FimH protein showing a mannose guest at the active site. Right: overlap of single crystal deoxy-mannose molecule [41] (stick display) and one of the 4 taurolidine molecules found in the unit cell. H atoms bound to C in deoxy-mannose are omitted for clarity.

The FimH crystal structure with a mannose bound at the active site on the surface of the protein [40], is shown in Figure 10 (code 1KLF in the Protein Databank). We superimpose the crystal structure of deoxy-mannose [41] with each of the 4 molecules found in the unit cell of taurolidine, the two independent molecules and their corresponding inverted molecules. A ring of one of these taurolidine molecules shows excellent overlap with mannose; this is also depicted in Figure 10.

**Figure 11:**
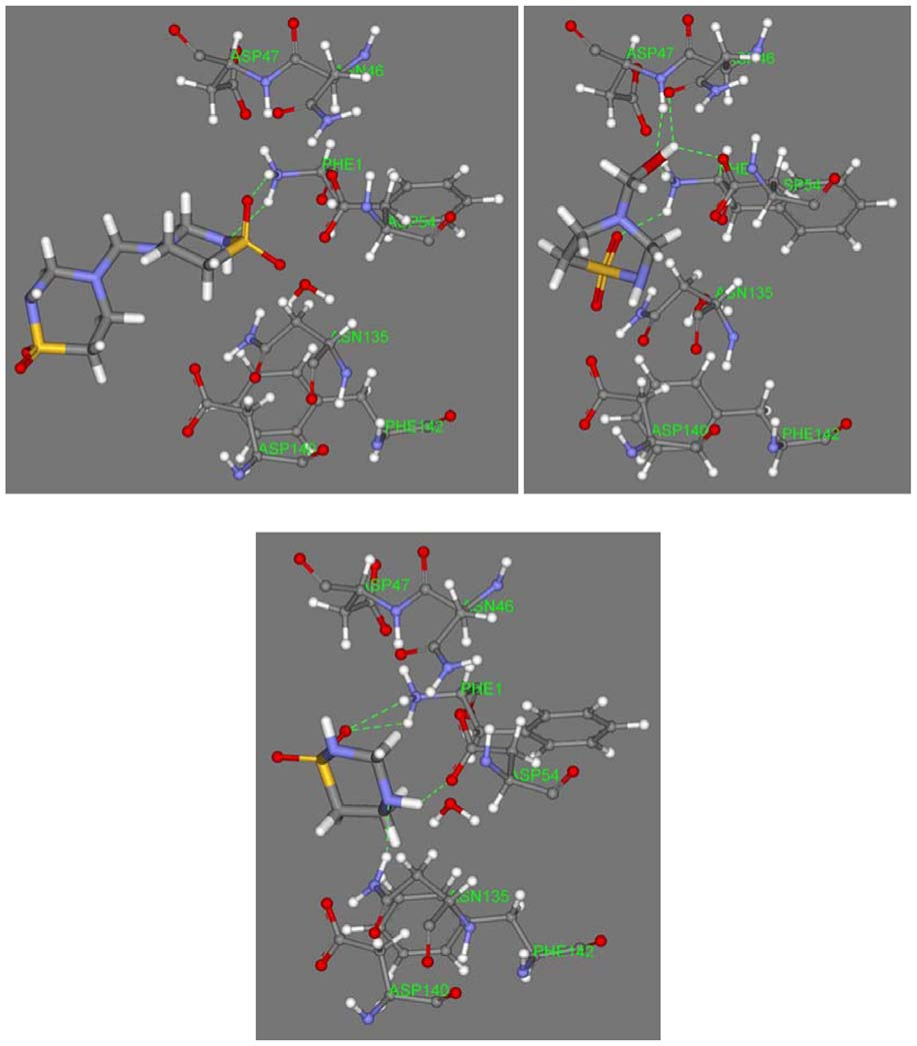
Stick display of taurolidine and its derivatives, methylol taurultam and taurultam, at the active site of *E. coli* FimH. Top left: taurolidine with H-bond interacting amino acids and water (ball and spoke display) shows the formation of a 6-membered ring -H-N(phe1)-H-O(sulfonyl)-S-N(taur)-; the non interacting taurolidine ring protrudes outside the protein pocket. Top right: methylol taurultam shows 4 H-bonds: (a) S(sulfonyl) as a hydrogen bond acceptor from the NH_3_-(Phe1), (b) O–H acts as a bifurcated hydrogen bond donor to O(carbonyl) of Arg46 and Phe1, (c) O(hydroxyl)as a hydrogen bond acceptor from HN-(Asp47). Bottom: taurultam also has 4 hydrogen bonds: (a) O(sulfonyl) as a hydrogen bond acceptor from two hydrogens of NH_3_-(Phe1), (b) N-H as hydrogen bond donor to O(carbonyl)-(Asp54) and (c) hydrogen bond acceptor from H-N-(Asn135).

**Figure 12:**
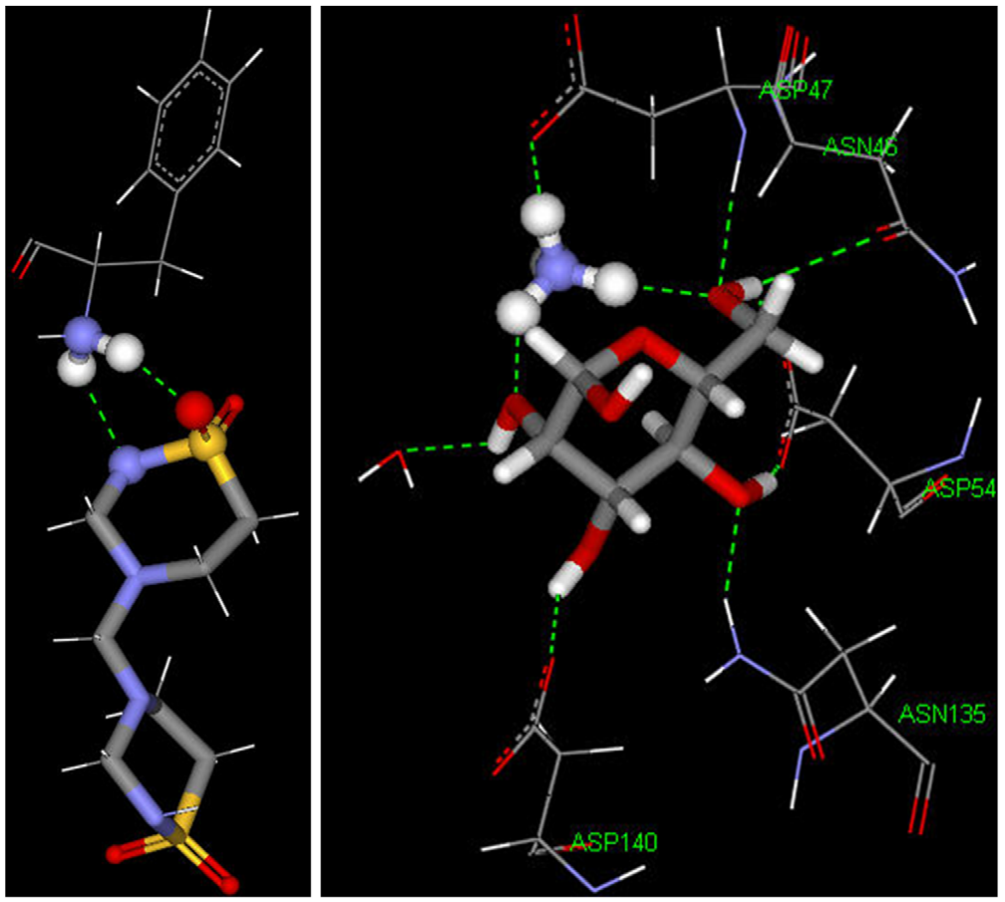
H-bond interaction of taurolidine and mannose at the FimH active site. Both molecules establish a double H-bond with NH_3_ of Phe1, e.g. a 6-membered ring for taurolidine (left, ball and stick style) and a 10-membered ring for mannose (left, C-NH_3_ moiety of Phe1, ball and stick style). For clarity, Phe1 is the only amino acid shown in the left figure (line style except for NH_2_), and only the C-NH_3_ moiety of Phe1 is shown for mannose on the right.

Docking of taurolidine, taurultam and methylol-taurultam at the fimbriae active site was performed after mannose exclusion from the protein crystal structure using the molecular mechanics program CDOCKER. After CHARMm force-field H generation [42], H coordinates at the active site were made consistent with the crystal structure [40]. Calculations for a non-zwitterionic polypeptide showed mannose with positive binding energy, indicating repulsion at the active site; therefore, we operated with the zwitterionic environment corresponding to a terminal (+)NH_3_ group for Phe1. Previous studies highlighted the importance of the amine terminal group after interaction of FimH with horseradish peroxidase, a highly mannosilated protein, and the active site of FimH was estimated in the range of aminoacids 1–100 [43]. For a first simulation, the active site was kept fixed and taurolidine, taurultam and methylol-taurultam in turn were allowed to fit. Ten poses were calculated for each guest and the binding energy, which describes guest affinity for the active site pocket, was calculated for each molecule's pose. Active site ligand minimizations, that included, in turn, all docked molecules, were performed later. Results are shown in Table 1 for the molecules having the best fit to the zwitterionic polypeptide; a corresponding calculation for the mannose guest is also included. Table 1, shows that taurolidine fits better than methylol-taurultam or taurultam as its binding energy is the most negative, −120.0, −96.9, and −58.6 kcal/mol, respectively. Interestingly, the affinity of taurolidine for the protein pocket is higher than that of mannose. In addition, electrostatic and van der Waals energy of mannose are repulsive (5.6 and 2.6 kcal/mol, respectively), whereas for taurolidine they are the most negative (−103.4 and −5.6 kcal/mol respectively) in Table 1; it is clear that the affinity of mannose for the active site of FimH is mainly due to its several H-bonds, Figure 11. In our docking studies, one ring of taurolidine protrudes outside the protein pocket, while for the ring inside, the O(sulfonyl) and the NH next to it, are acceptors to two H-bonds from the Phe1 [NH_3_]^+^ terminal group. This adjacent double H-bond interaction establishes a 6-membered ring -H-N(phe1)-H-O(sulfonyl)-S-N(taur)-, the first 3 atoms from the protein [NH_3_]^+^ moiety, and the remaining 3 atoms from taurolidine, Figure 12; one can assume that such cyclic arrangement is an important stabilization factor for taurolidine interaction with *E. coli*. This affinity may explain taurolidine's activity as an antibacterial drug after surgery, that is, the bacteria would prefer taurolidine instead of mannose in the epithelial tissue. The methylol-taurultam hydroxyl group forms H-bonds with FimH Phe1, Arg46, Asp47 and Asp54, whereas the O(sulfonyl) interacts with the [NH_3_]^+^ terminal group: again, there are 2 H from the [NH_3_]^+^ terminal group acting as H-bond donors, but they do not form a 6-membered ring as with taurolidine; one of its H points to Asp74. Taurultam also interacts with FimH through 4 H-bonds: the O(sulfonyl) forms H-bonds with 2 H from the [NH_3_]^+^ terminal group; two other H-bond interactions are due to the NH group far from SO_2_; they are formed with Asp54 and Asn135. Therefore, the 6-membered ring is exclusively formed by taurolidine. This arrangement is a major factor contributing to taurolidine binding energy and consequent affinity for FimH.

**Table 1:** Energy (kcal/mol) data for guests in the pocket of *E. coli* fimbriae protein active site.

	Mannose	Taurolidine	Methylol-taurultam	Taurultam
Binding energy	−107.7	−120.7	−96.9	−58.6
Electrostatic energy	5.6	−103.9	−53.8	−42.6
van der Waals energy	2.6	−5.6	−0.5	−2.0

The two-step decomposition pattern of taurolidine, Figure 1, shown earlier to stem from the H^+^ attack on taurolidine, implies that a proton transfer to the drug is needed for its hydrolysis. Taurolidine accumulates δ^+^ charge as a result of its interaction with Phe1[NH_3_]^+^ at the active site of FimH through formation of the double H-bond, as described above. We are investigating a potential decomposition mechanism of taurolidine at the active site to generate products shown in Figure 1.

### Conclusions

The crystal structure of taurolidine shows two independent molecules in the asymmetric unit with closely related internal energy (ΔE = 0.6 kcal/mol); this suggests taurolidine is a flexible molecule that is capable of conformational adaptation to the requisite geometries needed for biological activity. Crystal packing shows one of these two molecules somewhat positively charged due to asymmetric distribution of intermolecular H-bonds. The hydrolysis/decomposition pattern of taurolidine is described theoretically as a 2-step process, neither having an energy barrier. Methylol-taurultam hydrolysis probably follows a concerted mechanism using water as reagent. Our docking studies of binding energies show mannose more strongly bound than taurultam and methylol-taurultam at the active site of the protein whereas taurolidine shows the highest affinity, associated with a stable 6-membered ring. It is worth noting that taurolidine energy of interaction with fimbriae is not due to extensive H-bonding, thus making its electrostatic and van der Waals energies appear as a strong contributor to explain its affinity for the active site, as found by our docking results. Since taurolidine is readily available through its administration in high doses after peritonitis surgery it may effectively compete with mannose to avoid bacterial attack on laparoscopic lesions. Also, docking results suggest that the role of the hydrolysis taurolidine derivatives, taurultam and methylol-taurultam, appears less relevant than that of the parent compound. However, taurolidine application is also active against gram-positive bacteria and fungi, which do not display fimbriae proteins and, in such cases, a potential role for its hydrolysis products may be still possible. The [NH_3_]^+^ terminal group of Phe1 consistently forms H-bonds with taurolidine and all its derivatives; this effectively transfers a positive charge on taurolidine and is reminiscent of H^+^ necessary for the first step in taurolidine decomposition as calculated in this study.

Interaction of the taurolidine derivative methylol-taurultam with the diaminopimelic NH_2_ group in the bacteria cell wall has large negative ΔG but high activation energy and so little evidence for reactivity.

This high activation energy could be attenuated by appropriate (but so far, unknown) enzyme performance and this may induce reactivity; this process would imply a more general mechanism of action for taurolidine, i.e. both gram-negative and gram- positive bacteria would be affected. Moreover, peptidoglycan forms around 90% of the dry weight of gram-positive bacteria but only 10% of gram-negative, and so an effective reaction of methylol taurultam may be more significant on the gram-positive bacteria.

## Materials and Methods

The crystallographic study was performed using a Syntex-Crystallogic diffractometer using Mo radiation. The molecular structure was solved using direct methods [44] and refined using CAOS [45]. The crystalline title compound was provided by Carter-Wallace Inc. (Cranbury, NJ, USA). During data collection, some crystal decay was observed as the intensity of three standards ([2], [1], [3], [1,−1,3], [3,3,−1]) decreased constantly and anisotropically, −17.0%, −13.0% and −3.6%, respectively, see Table 2 for details.

**Table 2:** Crystal data and refinement details of taurolidine, C_7_H_16_N_4_O_4_S_2_.

M_w_	284.38
a (Å)	9.178(6)
b (Å)	10.892(7)
c (Å)	13.916(9)
α,β,γ	69.77(2), 87.36(2), 70.03(2)
V, Å^3^	1222.7(14)
Z	4
Crystal system, space group	Triclinic, P −1
Temp	293 K
Crystal size, mm, color	0.24×0.16×0.07,colorless
D_c_ g cm ^−3^	1.545
µ(Mo Kα) cm^−1^	0.446
absorption (ψ - scan), Tmin - Tmax	0.92–0.97
Diffractometer, method	Syntex Crystallogic 2θ - θ scans
X ray source	Fine focus sealed X-ray tube
Monochromator	Graphite
*h* min,max/*k* min,max/*l* min,max	−11,12/0,14/−16/18
θmin–θmax	1.56–28.09
Total no. reflections	6149
Unique reflections	5963
Reflections [I>3σI)]	3210
R (Rw)	0.0591 (0.0790)
Goodness of fit	0.88
Decay correction %	11.0
No. parameters refined	307
Highest/lowest difference Fourier peak	0.340/−0.987

The structural features, transition state and energy parameters for taurolidine and its derivatives were analyzed with theoretical methods using the density functional theory (DFT) program DMol3, implemented in the packages Cerius2 4.6 (SGI platform) and Materials Studio 4.4 (PC platform) from Accelrys [46]–[48]. The highest level of theory were used in these programs; in Cerius2 the local density was the Perdew and Wang (PWC) functional [49], in Materials Studio the local density setting was the general gradient approximation (GGA) and the Becke exchange (BP) functional [50]. A double numeric basis set with polarization functions (DNP) on all atoms was set in both DMol3 programs [51]. In addition, some calculations were performed with Hartree-Fock algorithms implemented in Spartan 06, v102 using 6–31G** basis set [52]. Taurolidine theoretical calculations were performed using initial coordinates from the corresponding X-ray molecular structure. All other molecules were *ab-initio* generated using DMol3.

Docking studies were performed with the molecular mechanics CDOCKER package in Discovery Studio 2.1 from Accelrys. CDOCKER is a grid-based molecular docking method that employs CHARMm [42]. We specified the ligand placement in the active site using a binding site sphere of radius 10 Å in the conformer simulation for each ligand. Structures of methylol taurultam, taurultam, and both molecules of taurolidine, stemming from coordinates obtained the crystal structure, were taken from the DFT calculations described above. They were used as initial configurations to define 10 poses for each molecule that were automatically placed in the active site by CDOCKER. Then, energy calculations were performed; in this initial process the receptor was held rigid. Several clusters of taurolidine confirmed its flexibility, already described from the crystal structure. Later, the binding site receptor was still held rigid while the ligands were allowed to flex during the refinement; the resulting binding energies along with specific van der Waals and electrostatic energies are shown in Table 1. Binding energy is the result of complex energy minus the addition of ligand energy plus receptor energy. In a final calculation ligand minimization was performed allowing the active site to be flexible, but the rest of the protein was held fixed.

## Supporting Information

Figure S1 Transition state search for taurolidine hydrolysis.
(1.68 MB TIF)

Table S1 Experimental structural parameters of taurolidine from the crystal.
(0.04 MB DOC)
