# Supplementary figures and images for: Taurolidine Antiadhesive Properties on Interaction with E. coli; Its Transformation in Biological Environment and Interaction with Bacteria Cell Wall
Source: PLoS One. 2010 Jan 28;5(1):e8927. doi: 10.1371/journal.pone.0008927 (PMC2812514; doi:10.1371/journal.pone.0008927)

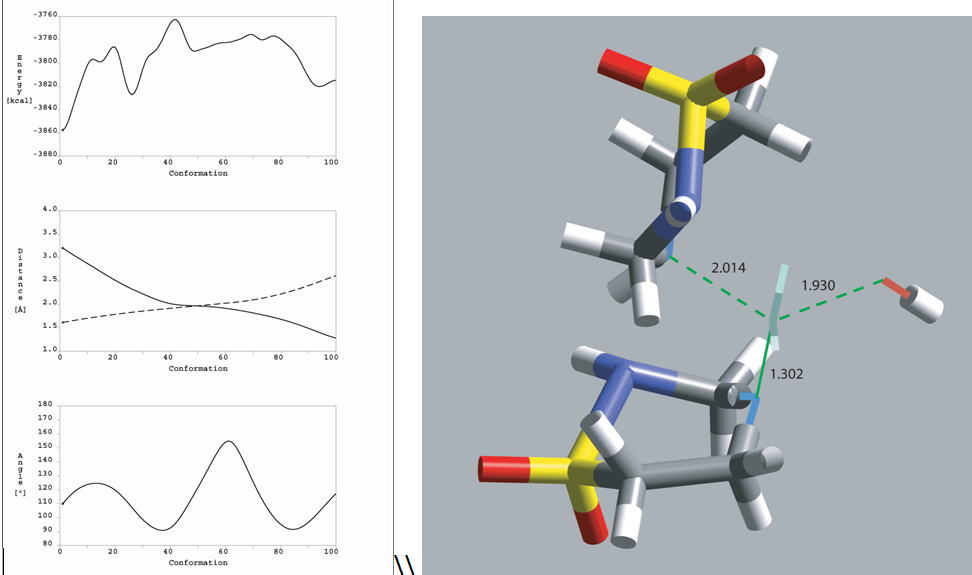

Supplement: Figure S1 — Transition state search for taurolidine hydrolysis. (1.68 MB TIF) [file pone.0008927.s001.tif]
