# Supplementary material for: Taurolidine Antiadhesive Properties on Interaction with E. coli; Its Transformation in Biological Environment and Interaction with Bacteria Cell Wall
Source: PLoS One. 2010 Jan 28;5(1):e8927. doi: 10.1371/journal.pone.0008927 (PMC2812514; doi:10.1371/journal.pone.0008927)
Supplement: Table S1 — Experimental structural parameters of taurolidine from the crystal. (0.04 MB DOC) [file pone.0008927.s002.doc]

Table S1. Experimental structural parameters of taurolidine from the crystal.

| Bond distances | | | |
| --- | --- | --- | --- |
| Molecule 1 | | Molecule 2 | |
| S1 O1 | 1.424(6) | S41 O41 | 1.436(6) |
| S1 O2 | 1.428(6) | S41 O42 | 1.430(6) |
| S1 N2 | 1.621(5) | S41 N42 | 1.620(6) |
| S1 C6 | 1.744(6) | S41 C46 | 1.742(7) |
| S21 O21 | 1.437(5) | S61 O61 | 1.435(5) |
| S21 O22 | 1.423(6) | S61 O62 | 1.428(5) |
| S21 N22 | 1.623(7) | S61 N62 | 1.615(5) |
| S21 C26 | 1.739(7) | S61 C66 | 1.752(8) |
| C7 N4 | 1.45(1) | C47 N44 | 1.453(8) |
| C7 N24 | 1.464(8) | C47 N64 | 1.452(8) |
| Bond angles | | | |
| O2 S1 O1 | 118.6(3) | O61 S61 O62 | 118.3(3) |
| N2 S1 O1 | 107.5(3) | N62 S61 O62 | 107.4(3) |
| N2 S1 O2 | 107.4(3) | N62 S61 O62 | 107.5(3) |
| C6 S1 O1 | 110.8(3) | C66 S61 O61 | 107.5(4) |
| C6 S1 O2 | 107.5(3) | C66 S61 O62 | 111.3(4) |
| C6 S1 N2 | 104.1(3) | C66 S61 N62 | 103.7(3) |
| N4 C7 N24 | 110.0(6) | N44 C47 N64 | 110.5(5) |
